# High-density Association Mapping and Interaction Analysis of *PLA2R1* and *HLA* Regions with Idiopathic Membranous Nephropathy in Japanese

**DOI:** 10.1038/srep38189

**Published:** 2016-12-09

**Authors:** Myo Thiri, Kenjiro Honda, Koichi Kashiwase, Akihiko Mabuchi, Hodaka Suzuki, Kimio Watanabe, Masaaki Nakayama, Tsuyoshi Watanabe, Kent Doi, Katsushi Tokunaga, Eisei Noiri

**Affiliations:** 1 Department of Human Genetics, Graduate School of Medicine, The University of Tokyo, Tokyo, Japan; 2 Department of Nephrology and Endocrinology, The University of Tokyo Hospital, Tokyo, Japan; 3 Japanese Red Cross Kanto-Koshinetsu Block Blood Center, Tokyo, Japan; 4 Department of Nephrology, Hypertension, Diabetology, Endocrinology and Metabolism, Fukushima Medical University School of Medicine, Fukushima, Japan; 5 Department of Hemodialysis and Apheresis, The University of Tokyo Hospital, Tokyo, Japan

## Abstract

Although recent studies showed anti-PLA2R antibody plays a crucial role in idiopathic membranous nephropathy (IMN), detailed *HLA* mapping and interaction between the *HLA* genes and *PLA2R1* have not been investigated in IMN. We genotyped across the *PLA2R*1 gene and the HLA region, using 183 IMN patients and 811 healthy controls. Five SNPs around the *PLA2R1* gene were significantly associated with IMN. In addition to the two SNPs previously reported to be strongly associated with IMN, rs3749119 and rs35771982 (OR 3.02 and 2.93, P = 3.24E-14 and 4.64E-14, respectively), two novel intronic SNPs (rs2715928 and rs16844715) were also identified as IMN-associated SNPs (OR = 2.30 and 2.51, P = 3.15E-10 and 5.66E-13, respectively). In the *HLA* gene analysis, DRB1*1501 and DQB1*0602 were strongly associated with IMN (P = 1.14E-11 and 1.25E-11, respectively). The interaction was strongest between HLA-DRB1*15:01 - HLA-DQB1*06:02 and the intronic SNP rs2715928 (OR = 17.53, P = 4.26E-26). Furthermore, positive interaction was also observed between HLA-DRB1*15:01 - HLA-DQB1*06:02 and the missense SNP rs35771982 (OR = 15.91, P = 2.76E-29), which is in strong linkage disequilibrium with 5′UTR SNP rs3749119, and intronic SNP rs16844715 (OR = 15.91, P = 2.30E-26) for IMN. Neither HLA-DRB1*15:01 nor HLA-DQB1*06:02 was associated with steroid responsiveness, overall survival and renal survival during the observation period of mean 11 years though limited number of analysis.

Idiopathic membranous nephropathy (IMN) is one of the most common causes of adult nephrotic syndrome. It is characterized by subepithelial immune complex deposition of glomerular basement membrane. The first antigen neural endopeptidase, type II transmembrane glycoprotein, was identified in neonatal membranous nephropathy (MN) cases with neural endopeptidase-deficient mothers^1^. The second autoantigen is the M-type phospholipase A_2_ receptor (PLA2R), identified in the adult patients with IMN^2^. PLA2R is found in the sera of 75% of IMN patients, but not in that of secondary MN or any other disorders of renal and autoimmune^3^.

Recent genome-wide association studies (GWAS) including three studies in European populations have identified that the variations in *PLA2R1* on chromosome 2 and *HLA-DQA1* on chromosome 6 show susceptibility to IMN^4^. Interestingly, Stanescu *et al*. also reported a strong genetic interaction of risk alleles at both the HLA and *PLA2R1* regions, although relatively modest risk of IMN was identified at each locus. Association of *HLA-DQA1* and *PLA2R1* with IMN was also reported by case-control candidate gene studies in Korea, Taiwan and China^5^,^6^,^7^.

In Japan, IMN was reported to account for 77.9% of total MN patients, while MN was present in 36.8% of primary nephrotic syndrome patients^8^. However, not many genetic studies of IMN in Japanese population have been conducted. Although previous studies have reported the effect of single locus and the genetic interaction of SNPs in *PLA2R1* and *HLA-DQA1*, no study has performed the high-density association mapping of both the *PLA2R1* gene and *HLA* genes to date for identifying the primary polymorphisms and it is still not known the interaction effect of *PLA2R1* risk variants and fully detailed analysis of *HLA*-gene alleles.

## Results

### *PLA2R1* association with IMN

#### Association analysis of *PLA2R1* SNPs in the first set of IMN patients and healthy controls

Fine mapping of *PLA2R1* SNPs was performed in the first stage of the study including 53 IMN patients and 419 healthy controls. The characteristics and clinical information of 53 IMN cases were described in Table 1. Of the 15 SNPs genotyped, two SNPs failed in genotyping and were excluded from the study. Significant deviations from Hardy-Weinberg equilibrium (P < 0.05) were not observed for any of the 13 SNPs. Of the13 SNPs included in the association analysis, we found 9 SNPs significantly associated with IMN (P < 0.05) (bold in Supplementary Table 1). When we corrected for the multiple testing, 7 SNPs survived to be significant (Table 2).

#### Replication in the second data set and combined analysis

Seven significant SNPs in the first set were attempted to replicate in a total of 130 IMN patients and 392 healthy controls. Five SNPs were successfully replicated in the second set.

When the association analysis was conducted in the combined data sets, rs35771982 and rs3749119 (which are in high linkage disequilibrium with each other) exhibited the strongest associations (P = 4.51E-15, OR = 2.93 and P = 1.99E-15, OR = 3.02, respectively) (Table 2). Two intronic SNPs, rs2715928 (P = 1.14E-10, OR = 2.30) and rs16844715 (P = 7.90E-13, OR = 2.51), also showed significant associations with IMN.

All the SNPs that were consistently significant in all data sets were selected in haplotype analysis. Analysis of linkage disequilibrium (LD) pattern showed high LD (r^2^ = 0.8) between rs35771982, missense SNP located in exon 5, and rs3749119 located in 5′ UTR region of *PLA2R1*. Risk haplotype composed of risk alleles (G for rs35771982, A for rs2715928 and C for rs16844715) exhibited strong association with IMN (P = 7.30E-13, OR = 2.29), while protective haplotype including protective alleles (C for rs35771982, G for rs2715928 and T for rs16844715) was found to be protective to IMN (P = 1.84E-14, OR = 0.34). (see Supplementary Table S2).

### Association of *HLA* genes with IMN

#### Association analysis of *HLA* genes in the first set of IMN patients and healthy controls

In the discovery stage by full detail analysis, *HLA-A, -B, -C, -DRB1, -DQB1* and *–DPB1* genotypes were determined in a total of 53 IMN patients and 419 healthy controls. Regarding *HLA* class I genes, HLA-A*33:03 showed marginal association (P = 0.03, OR = 0.40) with IMN (see Supplementary Table S3). Tendency of negative association was observed in HLA-B*07:02 with IMN while positive association was found with HLA-B*35:01 (see Supplementary Table S4). HLA-C*0704 exhibited a high odds ratio for IMN (P = 5.79E-03, OR = 5.89; see Supplementary Table S5). However, no alleles mentioned above remained significant after correction for multiple comparisons.

Regarding HLA class II genes, HLA-DRB1*15:01 was the most strongly associated allele (P = 7.72E-5, OR = 2.85), and it remained to be significant when P-value was corrected for the number of alleles tested (see Supplementary Table S6). Negative association was also observed in HLA-DRB1*04:05. On the other hand, four *HLA-DQB*1 alleles showed significant associations with IMN. Among them, HLA-DQB1*06:02 survived to be significant after correction for multiple comparisons (P = 5.12E-4, OR = 2.60) (see Supplementary Table S7). No association was observed between *HLA-DPB1* alleles and IMN (see Supplementary Table S8).

#### Replication in the second sample set and the combined set

The replication study of two *HLA* class II genes, *HLA-DRB1* and *HLA-DQB1*, was performed in an independent set of 130 IMN patients and 392 healthy controls. A significant positive association was observed for HLA-DRB1*15:01 and HLA-DQB1*06:02 with IMN in the replication stage with P = 1.71E-9, OR = 3.36 and P = 5.14E-10, OR = 3.56, respectively (Tables 3 and 4). Except for HLA-DRB1*13:02 and *HLA*-DQB1*06:04 which showed marginal association with IMN, none of the significant *HLA-DRB1* and *HLA-DQB1* alleles in first set was found to have P-value < 0.05.

In the combined data analysis, both HLA-DRB1*15:01 and HLA-DQB1*06:02 were confirmed to have strong associations with IMN holding P = 3.94E-13, OR = 3.09 and P = 8.90E-13, OR = 3.1, respectively (Tables 3 and 4). Both HLA-DRB1*15:01 and HLA-DQB1*06:02 remained significant after Bonferroni correction (P = 1.14E-11 and 1.25E-11, respectively).

#### *HLA DRB1-DQB1* haplotype association in the combined data set

The results of *HLA-DRB1-DQB1* haplotype association analysis were shown in Table 5. Only those haplotypes with frequencies >1% in either IMN patients or healthy controls were included in the analysis. The most susceptible haplotype was DRB1*15:01-DQB1*06:02 haplotype (P = 1.89E-12, OR = 3.07). Other susceptible haplotypes were DRB1*14:54-DQB1*05:02 haplotype (P = 2.67E-3, OR = 3.1), DRB1*11:01-DQB1*03:01 haplotype (P = 3.21E-3, OR = 2.34) and DRB1*04:01-DQB1*03:01 haplotype (P = 5.16E-3, OR = 2.71). The most protective haplotype was DRB1*13:02-DQB1*06:04 haplotype (P = 6.10E-3, OR = 0.44).

#### Genetic interaction analysis between *PLA2R1* and *HLA* risk alleles

We analyzed the total of 177 IMN cases and 792 healthy controls to investigate the interactions between HLA-DRB1*15:01-HLA-DQB1*06:02 and *PLA2R1* risk alleles.

Interaction analysis exhibited more than additive effects with IMN (Table 6). We observed that the evidence for the interaction was strongest between HLA-DRB1*15:01 - HLA-DQB1*06:02 and the intronic SNP rs2715928 (P = 4.26E-26, OR = 17.53). In addition, positive interaction was also observed between HLA-DRB1*15:01 - HLA-DQB1*06:02 and the missense SNP rs35771982 (P = 2.76E-29, OR = 15.91) that is in strong LD with 5′UTR SNP rs3749119, and intronic SNP rs16844715 (P = 2.30E-26, OR = 15.91) for IMN.

#### *HLA* and clinical outcome

During the observation period [median 11 years, interquartile range (IQR) 8.5–13 years], 50% increase of serum creatinine was found in eight patients. Among them, four patients developed to end stage renal disease. Ten patients died of heart failure, infection, and traffic accident. Supplementary Tables S9 and S10 shows overall survival and renal survival according to risk *HLA* alleles, HLA DRB1*15:01 and HLA DQB1*06:02. Neither risk alleles were associated with clinical outcome. Combination of risk alleles of *HLA* genes and *PLA2R1* were not associated with clinical outcome.

## Discussion

The genetic association of *PLA2R1* and *HLA-DQA1* risk alleles with IMN in the Caucasian populations was reported by a GWAS that included three independent GWAS in three populations^4^. Recent studies following this paper selected several SNPs as representative from *PLA2R1* and the HLA regions^7^,^9^,^10^,^11^. The diversity of *HLA* genes is well-known in human genetics and tightly bound to disease appearance and severity. Admittedly, it has been reported that IMN in Japanese has a better course and outcome^12^. The purpose of this study was to clarify: (i) the primary risk SNPs of *PLA2R1* in Japanese population, (ii) *HLA* typing in full detail, (iii) the interaction between the risk alleles of *PLA2R1* and *HLA* genes, and (iv) clinical outcome according to risk alleles.

We found 7 SNPs within *PLA2R1* gene confirmed to be significantly associated with IMN, including a non-synonymous SNP (H [His] ⇒ D [Asp]) and a 5′ UTR SNPs reported in previous studies and additional 5 SNPs. In Japanese population, rs35771982 of *PLA2R1* is reported to be most strongly associated with IMN. In agreement with Coenen *et al*.^9^ and Liu *et al*.^6^ we found that G allele of non-synonymous SNP rs35771982 (G/C) showed significantly increased risk of developing IMN. While Coenen *et al*.^9^ also reported that C allele of 5′UTR rs3749119 raised the risk of IMN, our data showed rs35771982 is in strong LD with 5′ UTR SNP rs3749119. Although Kim *et al*.^5^ reported that C allele of rs35771982 elevated the risk of IMN, our genotyping data is robust considering that we obtained the significant association of rs1511223 located in 3′ UTR with IMN which similar to a report by Saeed *et al*.^11^.

Interestingly, our study identified the new significant associations of two intronic SNPs, rs2715928 and rs16844715. Both are coincidentally located in the first intron of *PLA2R1*. Our findings suggest that risk haplotypes from the combination of common variants and SNP-SNP interaction within *PLA2R1* region may not explain the low occurrence of IMN in general population. In other words, *HLA* risk alleles possibly explain IMN development together with *PLA2R1* risk alleles.

With respect to *HLA* association with IMN, recent studies have reported the strong association of *HLA-DQA1* SNP rs2187668 with IMN in Caucasian and Chinese populations^4^,^7^. *HLA* is highly polymorphic region spanning approximately 7.6 megabase pairs (Mb) of genomic sequence on the short arm of chromosome 6^13^. It has been reported that rs2187668 is a tag SNP for HLA-DRB1*03:01 in northern European populations, and the haplotype including DRB1*03:01 was associated with MN^14^,^15^,^16^.

Our study identified the new significant association of HLA-DRB1*15:01 and DQB1*06:02 with IMN in Japanese population. These alleles are well known to form a common haplotype, HLA-DRB1*15:01-DQB1*06:02, in Japanese population^17^. No such association with IMN has been reported in other populations. Therefore, this is the first report of this *HLA* haplotype in IMN. Because associations of HLA-DRB1*15:01-DQB1*06:02 with various immune disorders have been reported in different populations^18^,^19^,^20^,^21^, it is conceivable that the determined haplotype should be functionally meaningful.

In a French population, increased frequency of *HLA-DR3* and decreased frequency of HLA-DMA*01:02 with IMN were observed^22^. In British IMN patients, Vaughan *et al*. reported the associations of HLA-DRB1*03:01, HLA-DQA1*05:01 and HLA-DQB1*02:01 with IMN^23^. However, both DRB1*03:01 and DQB1*02:01 alleles are not frequent in the Japanese population. Only limited studies have been reported regarding the *HLA* associations of IMN in worldwide until today.

*HLA-DRB1* could be associated with anti-PLA2R. Anti-PLA2R antibodies detected in 26 of 37 patients with IMN were reported to be IgG4 antibodies^2^. IgG4 co-localized with PLA2R in the immune complex deposition on the glomerular basement membrane in patients with IMN, but not in those with secondary MN. Significantly higher frequency of HLA-DRB1*15 was reported in primary sclerosing cholangitis patients with increased levels of IgG4^24^. *HLA* association with IgG4-related IMN has not been identified yet, and how or whether IgG4 may relate to IMN is still uncertain.

With respect to *HLA* class I, no association of *HLA* class I alleles with IMN has been reported to date. In our analysis of the discovery set, we observed a weak association of HLA-B*07:02 with IMN before correction appearing in 1 of 53 IMN patients (1.9%) and 53 of 419 healthy individuals (12.6%). Additionally, our results showed a weak association of HLA-C*01:02 with IMN before correction, although the association ceased to be significant after multiple correction. We also showed a potential risk of HLA-C*07:04 with Japanese IMN patients. HLA-C*07:04 was reported to increase relative risk for carbamazepine-induced cutaneous adverse reactions in Japanese^25^. These *HLA* class I alleles are expected to be assessed in larger number of samples.

Our results showed more than additive effects on the risk of IMN among the individuals with risk alleles of PLA2R1, HLA-DRB1*15:01 and DQB1*06:02. In patients with homozygous rs2715928 risk genotype AA, those who have risk alleles of both HLA-DRB1*15:01 and DQB1*06:02 developed approximately 3.03 times as frequently as those who have one risk allele with either HLA-DRB1*15:01 or DQB1*06:02. This finding may suggest that HLA-DRB1*15:01 and DQB1*06:02 regulate immunological response including PLA2R1 via CD4-positive T cells.

The associations among renal survival, overall survival, steroid responsiveness, and *HLA* genes were analyzed in this study. This is because the HLA region may explain that both mild severity of IMN in Japanese and the impact of PLA2R on clinical course of IMN. However, even the strongest association of HLA-DRB1*15:01 and DQB1*06:02 with IMN was not related to renal survival, overall survival and steroid responsiveness. The fact provided that HLA-DRB1*15:01 and DQB1*06:02 were only associated with IMN development and that they did not influence the better prognosis particularly observed in Japanese IMN patients. This means that HLA-DRB1*15:01 and -DQB1*06:02 are possibly the risk alleles in the other populations as well.

There are some limitations in the present study. Firstly, titers of PLA2R1 antibody was not measured because the DNA samples were obtained more than ten years ago and most of the patients were either deceased or unable for follow-up. However, median of observation period was as long as 11 years, which is sufficient to evaluate overall and renal survival though the number is small. In addition, although anti-PLA2R antibodies are found in Japanese IMN patients, the prevalence is lower than that of other countries^26^. Our report partially explains why PLA2R1 serum level is not so frequently increased in Japanese IMN. The second limitation is the relatively small sample number, because IMN is not a common disease. Japan Renal Biopsy Registry in 2009 and 2010 announced that annual occurrence of total MN is approximately 200^27^. The sample number of the present study is comparable to that of nationwide, and our results are statistically robust.

In summary, our study has identified *PLA2R1* risk variants, the *HLA* risk alleles and haplotypes for association with IMN in Japanese population. Our study is the first report to perform high-density association mapping including the *HLA* region and reveal the increased risk of interaction effect between *PLA2R1* risk variants and *HLA* risk haplotype in IMN. Individuals with *PLA2R1* and HLA-DRB1*15:01–DQB1*06:02 risk alleles have higher risk of developing IMN while they were not associated with overall and renal survival. We also found novel two intronic SNPs (rs2715928 and rs16844715) and confirmed previous findings of *PLA2R1* association with IMN.

## Methods

### Human subjects and sample collection

This study included a total of 994 subjects comprised of healthy controls and cases with (biopsy-proven) IMN. The study was approved by the Ethical Committees at The Faculty of Medicine at The University of Tokyo, BioBank Japan and the Pharma SNP Consortium (Tokyo, Japan) (http://www.jpma.or.jp/information/research/psc/e02psc/about.html). Written informed consent was obtained from each participant before sample collection. The first set of study included 53 biopsy-proven IMN patients from The Tokyo University Hospital and 419 healthy controls residing around Tokyo area. Diagnosis of the IMN cases was established by kidney biopsy together with other routine clinical procedures and the patients with malignancy, chronic infectious disease including hepatitis B and C viruses, and drug induced secondary MN were excluded from the study. The second set of study that included 130 IMN cases from BioBank Japan and 392 healthy controls was recruited for replication purposes from The Tokyo University Hospital and the Pharma SNP Consortium (Tokyo, Japan). Both the discovery and replication studies included the Japanese individuals.

### Single nucleotide polymorphism (SNP) selection of the *PLA2R1* gene

SNP genotype information of *PLA2R1* was downloaded from Phase III of the HapMap JPT population database (http://hapmap.ncbi.nlm.nih.gov/). HapMap data was analyzed using HaploView software (ver 4.1) and tag SNPs were selected by Tagger algorithm implemented in HaploView^28^. SNPs were chosen by applying the following selection criteria: i) minor allele frequency (MAF) threshold of >0.10 in the HapMap JPT population, ii) r^2^ threshold of greater than or equal to 0.8. A total of twelve tag SNPs meeting the criteria were selected together with additional three reported SNPs from previous literatures^29^, to validate their association with IMN in the Japanese population.

### Genotyping of *PLA2R1* SNPs

The genomic DNA was extracted from the peripheral blood at each institution following the standard protocol. The concentration of genomic DNA was determined using spectrophotometer (NanoDrop ND-1000, NanoDrop Technologies). Genotyping of the total 15 SNPs were performed using discovery sample set of 472 individuals by using the TaqMan SNP Genotyping Assay (ABI: Applied Biosystems Inc. Foster City, CA, USA) to determine the genotypes according to the manufacturer’s protocol. For TaqMan Genotyping Assay, 10 ng of genomic DNA was used per reaction well. The mixture for every reaction was prepared with 2.5 μl of KAPA PROBE FAST qPCR Master Mix (Kapa Biosystems, Woburn, MA), and 0.125 μl of TaqMan SNP Genotyping Assay primer/probe (40x) from Applied Biosystem and 1.375 μl of Milli Q water. Then, 4 μl of reaction mixture was added to the 1 μl of DNA template. The polymerase chain reaction (PCR) was performed using Light cycler 480II (Roche, Germany) with cycling parameters of 95 °C for 3 minutes, followed by 40 cycles of 95 °C for 15 seconds and 60 °C for 1 minute. TaqMan SNP genotyping of significant variants was also performed in the replication study including 522 IMN cases and healthy controls.

### HLA typing

Genotyping for six *HLA* genes (*HLA-A, -B, -C, -DRB1, -DQB1* and *-DPB1*) was performed in 53 Japanese IMN patients by the polymerase chain reaction (PCR)-Luminex typing method using the WAKFlow *HLA* typing kit (Wakunaga, Hirohsima, Japan). Briefly, target DNA was amplified by PCR (polymerase chain reaction) with biotinylated primers. The PCR product was then denatured and hybridized to complementary oligonucleotide probes immobilized on fluorescent coded microsphere beads. In the meanwhile, biotinylated PCR products were labeled with phycoerythrin-conjugated streptavidin and finally examined with Luminex 100. Genotype determination and data analysis was performed automatically using the WAKFlow typing software. For healthy control samples, we utilized *HLA* data published previously^30^. *HLA* typing of significant HLA regions, *HLA-DRB1* and -*DQB1* genes, was performed in the second set of samples for the replication study. *HLA-DQA1* alleles were excluded from this study because of its low frequency in Japanese population^31^.

All experiments were performed in accordance with approved guidelines and regulations.

### Statistical analysis

Samples with failed genotyping were removed from data analysis. To compare the allele and genotype frequencies between case and control groups, Chi-square test or Fisher’s exact test was applied as appropriate. In interaction analysis of *HLA* and *PLA2R1* risk variants, Chi-square test or Fisher’s exact test was also used. Departure from Hardy-Weinberg equilibrium was tested in all the SNPs by the chi-squared goodness-of-fit test. Association analyses of all the SNPs in *PLA2R1* gene were performed by using PLINK software (http://pngu.mgh.harvard.edu/purcell/plink/)^32^. HaploView software and the sliding window analysis implemented in PLINK were used to infer the linkage disequilibrium structure of *PLA2R1* SNPs and to perform a haplotype analysis of the *PLA2R1* gene. In the HLA association analysis, *HLA* allelic associations were analyzed by Chi-square or Fisher’s exact test. *HLA* haplotype association analysis was performed using Arlequin algorithm^33^. When any of the obtained value was zero, the odds ratio was calculated using Woolf’s correction. Bonferroni corrections were performed by standard method where P values were corrected for the number of alleles tested in each gene. More stringent multiple corrections using total number of 117 SNPs/alleles in *PLA2R1, HLA-DRB1* and *HLA-DQB1* were added when the SNPs/alleles were statistically significant in both sample sets.

## Additional Information

**How to cite this article**: Thiri, M. *et al*. High-density Association Mapping and Interaction Analysis of *PLA2R1* and *HLA* Regions with Idiopathic Membranous Nephropathy in Japanese. *Sci. Rep.* **6**, 38189; doi: 10.1038/srep38189 (2016).

**Publisher's note:** Springer Nature remains neutral with regard to jurisdictional claims in published maps and institutional affiliations.

## Supplementary Material

Supplementary Dataset

## Figures and Tables

**Table 1:** Patient Characteristics and clinical course of the discovery set (N = 53).

Age (yr)	61 ± 15
Sex (M:F)	30:23
sCre at KBx	0.92 ± 0.36
sCre after 1 yr	1.06 ± 0.74
sAlb at KBx	2.33 ± 0.44
sAlb after 1 yr	3.23 ± 0.95
Upro at KBx	6.28 ± 3.35
Upro after 1 yr	2.12 ± 2.66
Pathological stage	
I	8 (15%)
II	27 (51%)
III	12 (23%)
IV	0
N.A.	6 (11%)
Corticosteroid	28 (53%)
Cyclosporine	7 (13%)
Development to ESRD	4 (8%)
Death	8 (15%)
50% increase of sCre	8 (15%)
Improvement to Upro < 1 g/gCre	21 (40%)

Age (yr): Age at kidney biopsy. sCre at KBx: serum creatinine at kidney biopsy; sCre after 1 yr: serum creatinine one year after kidney biopsy; sAlb at KBx: serum albumin at kidney biopsy; sAlb after 1 yr: serum albumin one year after kidney biopsy; Upro at KBx: ratio of urine protein to creatinine (g/g) at kidney biopsy; Upro after 1 yr: ratio of urine protein to creatinine (g/g) one year after kidney biopsy; Development to ESRD: development to end stage renal disease during observation period; 50% increase of sCre: 50% increase of serum creatinine during observation period; Improvement to Upro <1 g/gCre: improvement to ratio of urine protein to creatinine (g/g) less than 1.

**Table 2:** Replication and Combined analysis of significant *PLA2R1* SNPs.

SNP	Position	Risk allele	Replication analysis						Combined analysis					
			Case (n = 130)		Control (n = 386)		OR (95% CI)^a^	P-value^b^ corrected	Case (n = 183)		Control (n = 805)		OR (95% CI)^a^	P-value^b^ corrected
			No	%	No	%			No	%	No	%		
**rs1511223**	**3′ UTR**	A	203	79.3	536	70.9	1.57 (1.12–2.21)	1.57E-01	293	80.5	1105	69.7	**1.80 (1.36–2.38)**	**1.08E-03**
**rs35771982**	**H [His] ⇒ D [Asp]**	G	200	78.7	450	59.1	2.57 (1.84–3.58)	1.88E-08	289	79.8	919	57.5	**2.93 (2.22–3.85)**	**4.51E-15**
**rs10196882**	**intronic**	T	52	20.8	119	15.7	1.41 (0.98–2.02)	N.S.	82	23.0	245	15.5	**1.63 (1.23–2.16)**	**2.95E-02**
**rs877635**	**intronic**	A	69	27.2	200	26.5	1.03 (0.75–1.42)	N.S.	123	34.0	403	25.3	**1.52 (1.19–1.94)**	**4.78E-02**
**rs2715928**	**intronic**	A	185	71.7	385	51.7	2.36 (1.74–3.21)	4.56E-07	251	70.1	775	50.5	**2.30 (1.79–2.94)**	**1.14E-10**
**rs16844715**	**intronic**	C	168	66.1	350	46.7	2.23 (1.66–3.00)	5.16E-07	245	68.1	728	46.0	**2.51 (1.97–3.19)**	**7.90E-13**
**rs3749119**	**5**′ **UTR**	C	198	79.2	453	59.3	2.61 (1.86–3.66)	1.63E-08	286	80.8	929	58.2	**3.02 (2.28–4.01)**	**1.99E-15**

^a^OR: Odds ratio; 95% CI: lower and upper limits of confidence interval at 95%.

^b^P-values after Bonferroni correction and age/sex adjustment for allele frequency comparisons between cases and controls using the chi-square test^8^.

**Table 3:** Replication and Combined analysis of significant *HLA-DRB1* alleles.

*HLA-DRB1* alleles	Replication analysis							Combined analysis						
	IMN (2n = 258)		Control (2n = 772)		OR (95% CI)^a^	P-value^b^	P-value^c^ corrected	IMN (2n = 364)		Control (2n = 1610)	%	OR (95% CI)^a^	P-value^b^	P-value^c^ corrected
	No	%	No	%				No	%	No				
DRB1*0101	11	4.3	59	7.6	0.54 (0.28–1.04)	0.06	N.S.	13	3.6	116	7.2	0.48 (0.27–0.86)	0.01	0.33
DRB1*0401	9	3.5	11	1.4	2.50 (1.02–6.10)	0.03	0.77	12	3.3	21	1.3	2.58 (1.26–5.29)	7.42E-03	0.22
DRB1*0405	24	9.3	79	10.2	0.90 (0.56–1.45)	0.67	N.S.	31	8.5	201	12.5	0.65 (0.44–0.97)	0.03	0.98
DRB1*1101	12	4.7	15	1.9	2.46 (1.14–5.33)	0.02	0.53	18	4.9	38	2.4	2.15 (1.21–3.82)	7.31E-03	0.21
DRB1*1302	9	3.5	63	8.2	0.41 (0.20–0.83)	3.49E-03	0.10	12	3.3	128	8.0	0.39 (0.22–0.72)	1.79E-03	0.05
DRB1*1454	16	6.2	23	3.0	2.15 (1.12–4.14)	0.02	0.55	22	6.0	49	3.0	2.05 (1.22–3.43)	5.50E-03	0.16
DRB1*1501	52	20.2	54	7.0	3.36 (2.22–5.06)	1.71E-09	**4.97E-08**	73	20.1	121	7.5	3.09 (2.25–4.24)	3.94E-13	**1.14E-11**

^a^OR: Odds ratio; 95% CI: lower and upper limits of confidence interval at 95%.

^b^P-value for allele or genotype frequency comparisons between cases and controls using the chi-square test.

^c^P-value: Corrected P-value for the number of alleles tested.

**Table 4:** Replication and Combined analysis of significant *HLA-DQB1* alleles.

*HLA-DQB1* alleles	Replication analysis							Combined analysis						
	IMN (n = 258)		Control (n = 772)		OR (95% CI)^a^	P-value^b^	P-value^c^ corrected	IMN (n = 364)		Control (n = 1610)		OR (95% CI)^a^	P-value^b^	P-value^c^ corrected
	No	%	No	%				No	%	No	%			
DQB1*0401	23	8.9	76	9.8	0.90 (0.55–1.46)	0.66	N.S.	30	8.2	198	12.3	0.64 (0.43–0.96)	0.03	0.40
DQB1*0501	11	4.3	60	7.8	0.53 (0.27–1.02)	0.05	N.S.	14	3.8	123	7.6	0.48 (0.27–0.85)	0.01	0.14
DQB1*0502	11	4.3	14	1.8	2.41 (1.08–5.38)	0.03	0.38	16	4.4	31	1.9	2.34 (1.27–4.33)	5.24E-03	0.07
DQB1*0503	16	6.2	32	4.1	1.53 (0.82–2.83)	0.17	N.S.	23	6.3	62	3.9	1.68 (1.03–2.76)	0.04	N.S.
DQB1*0602	51	19.8	50	6.5	3.56 (2.34–5.41)	5.14E-10	**7.20E-09**	70	19.2	115	7.1	3.10 (2.24–4.27)	8.90E-13	**1.25E-11**
DQB1*0604	9	3.5	54	7.0	0.48 (0.23–0.99)	0.01	0.20	12	3.3	117	7.3	0.44 (0.24–0.80)	5.64E-03	0.08

^a^OR: Odds ratio; 95% CI: lower and upper limits of confidence interval at 95%.

^b^P-value for allele or genotype frequency comparisons between cases and controls using the chi-square test.

^c^P-value: Corrected P-value for the number of alleles tested.

**Table 5:** Frequency distribution of DRB1-DQB1 haplotypes in Japanese IMN patients and controls.

*DRB1-DQB1* haplotypes	IMN		Control		OR (95% CI)^a^	P-value^b^
	No	%	No	%		
DRB1*01:01-DQB1*05:01	13	7.1%	115	14.4%	0.48 (0.27–0.86)	**0.01**
DRB1*04:01-DQB1*03:01	12	6.6%	20	2.5%	2.71 (1.31–5.59)	**5.16E-03**
DRB1*04:03-DQB1*03:02	6	3.3%	46	5.8%	0.57 (0.24–1.34)	0.07
DRB1*04:05-DQB1*04:01	30	16.5%	194	24.3%	0.65 (0.44–0.98)	**0.04**
DRB1*04:06-DQB1*03:02	14	7.7%	61	7.6%	1.01 (0.56–1.83)	0.96
DRB1*04:10-DQB1*04:02	2	1.1%	21	2.6%	0.42 (0.1–1.79)	0.12
DRB1*08:02-DQB1*03:02	7	3.8%	33	4.1%	0.94 (0.41–2.13)	0.16
DRB1*08:02-DQB1*04:02	5	2.7%	28	3.5%	0.79 (0.3–2.05)	0.17
DRB1*08:03-DQB1*06:01	18	9.9%	124	15.5%	0.62 (0.37–1.03)	0.06
DRB1*09:01 DQB1*03:03	35	19.2%	222	27.8%	0.66 (0.46–0.97)	**0.03**
DRB1*10:01 DQB1*05:01	1	0.5%	8	1.0%	0.55 (0.07–4.42)	0.32
DRB1*11:01 DQB1*03:01	18	9.9%	35	4.4%	2.34 (1.31–4.17)	**3.21E-03**
DRB1*12:01 DQB1*03:01	7	3.8%	37	4.6%	0.83 (0.37–1.88)	0.15
DRB1*12:01 DQB1*03:03	4	2.2%	10	1.3%	1.77 (0.55–5.69)	0.15
DRB1*12:02 DQB1*03:01	3	1.6%	27	3.4%	0.49 (0.15–1.61)	0.10
DRB1*13:01 DQB1*06:03	3	1.6%	8	1.0%	1.66 (0.44–6.29)	0.20
DRB1*13:02 DQB1*06:04	12	6.6%	116	14.5%	0.44 (0.24–0.8)	**6.10E-03**
DRB1*13:02 DQB1*06:09	0	0.0%	11	1.4%	0.19 (0.01–3.24)	0.11
DRB1*14:03 DQB1*03:01	4	2.2%	27	3.4%	0.65 (0.23–1.87)	0.15
DRB1*14:05 DQB1*05:03	11	6.0%	29	3.6%	1.7 (0.84–3.43)	0.14
DRB1*14:06 DQB1*03:01	9	4.9%	25	3.1%	1.6 (0.74–3.47)	0.08
DRB1*14:54 DQB1*05:02	11	6.0%	16	2.0%	3.1 (1.43–6.73)	**2.67E-03**
DRB1*14:54 DQB1*05:03	11	6.0%	32	4.0%	1.53 (0.77–3.07)	0.22
DRB1*15:01 DQB1*03:01	4	2.2%	2	0.3%	8.92 (1.63–48.87)	**0.01**
DRB1*15:01 DQB1*06:02	69	37.9%	114	14.3%	3.07 (2.22–4.24)	**1.89E-12**
DRB1*15:02 DQB1*06:01	42	23.1%	172	21.5%	1.09 (0.76–1.56)	0.64
DRB1*16:02 DQB1*05:02	5	2.7%	9	1.1%	2.47 (0.82–7.42)	0.07

Haplotypes with frequencies of <1% in both cases and controls are omitted.

^a^OR: Odds ratio; 95% CI: lower and upper limits of confidence interval at 95%.

^b^P-value for allele or genotype frequency comparisons between cases and controls using the chi-square test.

**Table 6:** Interaction analysis between HLA-DRB1*15:01-DQB1*06:02 haplotype and *PLA2R1* risk variants.

*HLA*	*PLA2R1*	IMN (N = 177)		Controls (N = 792)		OR (95%CI)	P-value
		No	%	No	%		
DRB1*15:01-DQB1*06:02	rs1511223: A/A						
+	+	45	25.4	44	5.5	9 (5.32–15.24)	1.25E-19
+	−	17	9.6	65	8.2	2.3 (1.23–4.3)	7.44E-03
−	+	74	41.8	323	40.7	2.02 (1.34–3.04)	6.78E-04
−	−	41	23.2	360	45.5	1	
DRB1*15:01-DQB1*06:02	rs35771982: G/G						
+	+	41	23.2	27	3.4	15.91 (8.94–28.3)	2.76E-29
+	−	21	11.9	82	10.4	2.68 (1.52–4.75)	4.74E-04
−	+	71	40.1	222	28.0	3.35 (2.23–5.04)	1.78E-09
−	−	44	24.9	461	58.2	1	
DRB1*15:01-DQB1*06:02	rs2715928: A/A						
+	+	33	18.6	15	1.9	17.53 (9.03–34.03)	4.26E-26
+	−	29	16.4	94	11.9	2.46 (1.5–4.02)	2.35E-04
−	+	51	28.8	173	21.8	2.35 (1.56–3.53)	2.68E-05
−	−	64	36.2	510	64.4	1	
DRB1*15:01-DQB1*06:02	rs16844715: C/C						
+	+	34	19.2	18	2.3	15.91 (8.49–29.79)	2.30E-26
+	−	28	15.8	91	11.5	2.59 (1.58–4.26)	1.13E-04
−	+	51	28.8	144	18.2	2.98 (1.98–4.5)	1.98E-07
−	−	64	36.2	539	68.1	1	
DRB1*15:01-DQB1*06:02	rs3749119: C/C						
+	+	41	23.2	27	3.4	15.86 (8.9–28.26)	6.42E-29
+	−	21	11.9	82	10.4	2.67 (1.51–4.74)	5.21E-04
−	+	72	40.7	234	29.5	3.21 (2.13–4.84)	7.29E-09
−	−	43	24.3	449	56.7	1	
