# Supplementary material for: High-density Association Mapping and Interaction Analysis of PLA2R1 and HLA Regions with Idiopathic Membranous Nephropathy in Japanese
Source: Sci Rep. 2016 Dec 9;6:38189. doi: 10.1038/srep38189 (PMC5146917; doi:10.1038/srep38189)
Supplement: Supplementary Dataset [file srep38189-s1.doc]

High-density Association Mapping and Interaction Analysis of *PLA2R1* and *HLA* Regions with Idiopathic Membranous Nephropathy in Japanese

Myo Thiri^1^, Kenjiro Honda^2^, Koichi Kashiwase^3^, Akihiko Mabuchi^1^, Hodaka Suzuki^4^, Kimio Watanabe^4^, Masaaki Nakayama^4^, Tsuyoshi Watanabe^4^, Kent Doi^2^, Katsushi Tokunaga^1^, Eisei Noiri^2,5^

^1^ Department of Human Genetics, Graduate School of Medicine, The University of Tokyo, Tokyo, Japan

^2^Department of Nephrology and Endocrinology, The University of Tokyo Hospital, Tokyo, Japan

^3^Japanese Red Cross Kanto-Koshinetsu Block Blood Center, Tokyo, Japan

^4^Department of Nephrology, Hypertension, Diabetology, Endocrinology and Metabolism, Fukushima Medical University School of Medicine

^5^Department of Hemodialysis and Apheresis, The University of Tokyo Hospital, Tokyo, Japan

Supplementary Table S1. Association analysis of *PLA2R1* SNPs with IMN in the first sample set

| SNP | Position | Allele | Case (2n=106) | |  | Control (2n=838) | | OR (95% CI)^a^ | P-value^b^ |
| --- | --- | --- | --- | --- | --- | --- | --- | --- | --- |
|  |  |  | No | % |  | No | % |  |  |
| **rs1511223** | **3' UTR** | A | 88 | 83.0 |  | 569 | 68.6 | **2.24(1.32-3.8)** | **3.08E-03** |
|  |  | C | 18 | 17.0 |  | 261 | 31.4 |  |  |
| rs2715931 | intronic | A | 18 | 17.0 |  | 94 | 11.2 | 1.61(0.93-2.8) | 0.13 |
|  |  | C | 88 | 83.0 |  | 742 | 88.8 |  |  |
| **rs35771982** | **missense** | G | 87 | 82.1 |  | 469 | 56.1 | **3.58(2.14-6)** | **2.99E-07** |
|  |  | C | 19 | 17.9 |  | 367 | 43.9 |  |  |
| rs10929964 | intronic | T | 27 | 26.0 |  | 201 | 24.3 | 1.09(0.69-1.74) | 0.30 |
|  |  | A | 77 | 74.0 |  | 627 | 75.7 |  |  |
| **rs2203053** | **intronic** | G | 55 | 52.9 |  | 345 | 41.5 | **1.58(1.05-2.39)** | **6.17E-03** |
|  |  | A | 49 | 47.1 |  | 487 | 58.5 |  |  |
| **rs10196882** | **intronic** | T | 30 | 28.8 |  | 126 | 15.3 | **2.25(1.42-3.58)** | **4.14E-03** |
|  |  | C | 74 | 71.2 |  | 700 | 84.7 |  |  |
| rs6751162 | intronic | A | 24 | 23.1 |  | 181 | 22 | 1.07(0.66-1.73) | 0.39 |
|  |  | C | 80 | 76.9 |  | 643 | 78 |  |  |
| **rs16844706** | **intronic** | C | 42 | 43.7 |  | 275 | 33.4 | **1.55(1.01-2.38)** | **8.27E-03** |
|  |  | T | 54 | 56.3 |  | 549 | 66.6 |  |  |
| rs4665143 | synonymous | A | 43 | 42.2 |  | 308 | 37.1 | 1.24(0.81-1.88) | 0.18 |
|  |  | G | 59 | 57.8 |  | 522 | 62.9 |  |  |
| **rs877635** | **intronic** | A | 52 | 49.1 |  | 199 | 23.9 | **3.07(2.03-4.64)** | **1.10E-06** |
|  |  | C | 54 | 50.9 |  | 635 | 76.1 |  |  |
| **rs2715928** | **intronic** | A | 66 | 67.3 |  | 390 | 49.4 | **2.12(1.36-3.3)** | **5.84E-04** |
|  |  | G | 32 | 32.7 |  | 400 | 50.6 |  |  |
| **rs16844715** | **intronic** | C | 75 | 72.1 |  | 378 | 45.3 | **3.12(1.99-4.89)** | **6.21E-07** |
|  |  | T | 29 | 27.9 |  | 456 | 54.7 |  |  |
| **rs3749119** | **5' UTR** | C | 86 | 84.3 |  | 476 | 57.2 | **4.02(2.32-6.97)** | **7.02E-08** |
|  |  | T | 16 | 15.7 |  | 356 | 42.8 |  |  |

^a^OR: Odds ratio; 95% CI: lower and upper limits of confidence interval at 95%.

^b^P-values after age/sex adjustment for allele frequency comparisons between cases and controls using the chi-square test.

Supplementary Table S2. Haplotype association analysis of *PLA2R1* SNPs in the combined sample set

| Haplotype | Effect | rs35771982 | rs2715928 | rs16844715 | Case freq (n=183) | Control freq (n=805) | OR (95% CI) | P-value |
| --- | --- | --- | --- | --- | --- | --- | --- | --- |
| **H1** | **Risk** | **G** | **A** | **C** | 0.573 | 0.368 | **2.29 (1.65-3.17)** | **7.30E-13** |
| **H2** | **Protective** | **C** | **G** | **T** | 0.191 | 0.406 | **0.34 (0.23-0.5)** | **1.84E-14** |
| H3 | Neutral | G | G | C | 0.106 | 0.076 | _ | 0.06 |
| H4 | Neutral | G | A | T | 0.121 | 0.121 | _ | 1.00 |

| *HLA-A* | Allele frequency | | | | | |
| --- | --- | --- | --- | --- | --- | --- |
| alleles^a^ | IMN (2n=106) | | Control (2n=838) | | OR (95% CI)^b^ | P-value^c^ |
|  | No | % | No | % |  |  |
| *A*0101* | 0 | 0.0 | 10 | 1.2 | 0.37(0.02-6.38) | 0.30 |
| *A*0201* | 17 | 16.0 | 85 | 10.1 | 1.70(0.96-2.99) | 0.06 |
| *A*0206* | 8 | 7.5 | 61 | 7.3 | 1.04(0.48-2.24) | 0.15 |
| *A*0207* | 3 | 2.8 | 23 | 2.7 | 1.03(0.31-3.51) | 0.24 |
| *A*1101* | 11 | 10.4 | 80 | 9.5 | 1.10(0.57-2.14) | 0.78 |
| *A*2402* | 36 | 34.0 | 316 | 37.7 | 0.85(0.56-1.31) | 0.46 |
| *A*2420* | 0 | 0.0 | 10 | 1.2 | 0.37(0.02-6.38) | 0.30 |
| *A*2601* | 7 | 6.6 | 67 | 8.0 | 0.82(0.36-1.83) | 0.14 |
| *A*2602* | 3 | 2.8 | 12 | 1.4 | 2.01(0.56-7.24) | 0.15 |
| *A*2603* | 5 | 4.7 | 22 | 2.6 | 1.84(0.68-4.97) | 0.10 |
| *A*3101* | 12 | 11.3 | 67 | 8.0 | 1.47(0.77-2.82) | 0.24 |
| ***A*3303*** | 4 | 3.8 | 76 | 9.1 | **0.39(0.14-1.10)** | **0.03** |

^a^alleles: risk alleles with frequencies of less than 1% in both cases and controls are omitted.

Supplementary Table S3. Association analysis of *HLA-A* alleles with IMN in the first set

^b^OR: Odds ratio; 95% CI: lower and upper limits of confidence interval at 95%.

^c^P-values for allele frequency comparisons between cases and controls using the chi-square test.

^a^alleles: risk alleles with frequencies of less than 1% in both cases and controls are omitted.

Supplementary Table S4. Association analysis of *HLA-B* alleles with IMN in the first set

| *HLA-B* | Allele frequency | | | | | |
| --- | --- | --- | --- | --- | --- | --- |
| alleles^a^ | IMN (2n=106) | | Control (2n=838) | | OR (95% CI)^b^ | P-value^c^ |
|  | No | % | No | % |  |  |
| ***B*0702*** | 1 | 0.9 | 57 | 6.8 | **0.13(0.02-0.95)** | **6.33E-03** |
| *B*1301* | 2 | 1.9 | 13 | 1.6 | 1.22(0.27-5.50) | 0.28 |
| *B*1501* | 15 | 14.2 | 71 | 8.5 | 1.79(0.98-3.25) | 0.05 |
| *B*1518* | 4 | 3.8 | 14 | 1.7 | 2.31(0.75-7.16) | 0.09 |
| ***B*3501*** | 15 | 14.2 | 67 | 8.0 | **1.90(1.04-3.47)** | **0.03** |
| *B*3901* | 3 | 2.8 | 34 | 4.1 | 0.69(0.21-2.29) | 0.19 |
| *B*4001* | 5 | 4.7 | 46 | 5.5 | 0.85(0.33-2.20) | 0.18 |
| *B*4002* | 12 | 11.3 | 57 | 6.8 | 1.75(0.91-3.39) | 0.09 |
| *B*4006* | 4 | 3.8 | 34 | 4.1 | 0.93(0.32-2.67) | 0.21 |
| ***B*4403*** | 3 | 2.8 | 68 | 8.1 | **0.33(0.10-1.07)** | **0.02** |
| *B*4601* | 4 | 3.8 | 38 | 4.5 | 0.83(0.29-2.37) | 0.20 |
| *B*4801* | 3 | 2.8 | 22 | 2.6 | 1.08(0.32-3.68) | 0.24 |
| *B*5101* | 8 | 7.5 | 71 | 8.5 | 0.88(0.41-1.89) | 0.15 |
| *B*5201* | 12 | 11.3 | 80 | 9.5 | 1.21(0.64-2.31) | 0.56 |
| *B*5401* | 5 | 4.7 | 65 | 7.8 | 0.59(0.23-1.50) | 0.09 |
| *B*5502* | 0 | 0.0 | 20 | 2.4 | 0.19(0.01-3.13) | 0.09 |
| *B*5901* | 1 | 0.9 | 16 | 1.9 | 0.49(0.06-3.74) | 0.28 |
| *B*6701* | 3 | 2.8 | 11 | 1.3 | 2.20(0.60-8.00) | 0.14 |

^b^OR: Odds ratio; 95% CI: lower and upper limits of confidence interval at 95%.

^c^P-values for allele frequency comparisons between cases and controls using the chi-square test.

| *HLA-C* | Allele frequency | | | | | |
| --- | --- | --- | --- | --- | --- | --- |
| alleles^a^ | IMN (2n=106) | | Control (2n=838) | | OR (95% CI)^b^ | P-value^c^ |
|  | No | % | No | % |  |  |
| ***Cw*0102*** | 9 | 8.5 | 139 | 16.6 | **0.47(0.23-0.95)** | **0.01** |
| *Cw*0303* | 22 | 20.8 | 113 | 13.5 | 1.68(1.01-2.80) | 0.04 |
| *Cw*0304* | 16 | 15.1 | 105 | 12.5 | 1.24(0.70-2.20) | 0.45 |
| *Cw*0401* | 6 | 5.7 | 42 | 5.0 | 1.14(0.47-2.75) | 0.17 |
| *Cw*0702* | 10 | 9.4 | 121 | 14.4 | 0.62(0.31-1.22) | 0.16 |
| ***Cw*0704*** | 5 | 4.7 | 7 | 0.8 | **5.89(1.84-18.91)** | **5.79E-03** |
| *Cw*0801* | 8 | 7.5 | 48 | 5.7 | 1.35(0.62-2.93) | 0.12 |
| *Cw*0803* | 3 | 2.8 | 12 | 1.4 | 2.01(0.56-7.24) | 0.15 |
| *Cw*1202* | 12 | 11.3 | 81 | 9.7 | 1.20(0.63-2.28) | 0.58 |
| *Cw*1402* | 5 | 4.7 | 50 | 6.0 | 0.78(0.30-2.01) | 0.16 |
| ***Cw*1403*** | 3 | 2.8 | 69 | 8.2 | **0.33(0.10-1.05)** | **0.02** |
| *Cw*1502* | 5 | 4.7 | 31 | 3.7 | 1.29(0.49-3.40) | 0.17 |

^a^alleles: risk alleles with frequencies of less than 1% in both cases and controls are omitted.

Supplementary Table S5. Association analysis of *HLA-C* alleles with IMN in the first set

^b^OR: Odds ratio; 95% CI: lower and upper limits of confidence interval at 95%.

^c^P-values for allele frequency comparisons between cases and controls using the chi-square test.

| Supplementary Table S6. Association analysis of *HLA-DRB1* alleles with IMN in the first set | | | | | |  |
| --- | --- | --- | --- | --- | --- | --- |
| *HLA-DRB1* | Allele frequency | | | | | |
| alleles^a^ | IMN (2n=106) | | Control (2n=838) | | OR (95% CI)^b^ | P-value^c^ |
|  | No | % | No | % |  | corrected |
| ***DRB1*0101*** | 2 | 1.9 | 57 | 6.8 | **0.26(0.06-1.10)** | **0.02** |
| *DRB1*0401* | 3 | 2.8 | 10 | 1.2 | 2.42(0.65-8.93) | 0.12 |
| *DRB1*0403* | 2 | 1.9 | 24 | 2.9 | 0.65(0.15-2.81) | 0.24 |
| ***DRB1*0405*** | 7 | 6.6 | 122 | 14.6 | **0.42(0.19-0.92)** | **0.01** |
| *DRB1*0406* | 5 | 4.7 | 28 | 3.3 | 1.44(0.54-3.80) | 0.15 |
| *DRB1*0410* | 1 | 0.9 | 12 | 1.4 | 0.66(0.08-5.11) | 0.35 |
| *DRB1*0802* | 5 | 4.7 | 32 | 3.8 | 1.25(0.48-3.28) | 0.18 |
| *DRB1*0803* | 6 | 5.7 | 63 | 7.5 | 0.74(0.31-1.75) | 0.13 |
| *DRB1*0901* | 13 | 12.3 | 128 | 15.3 | 0.78(0.42-1.43) | 0.42 |
| *DRB1*1101* | 6 | 5.7 | 23 | 2.7 | 2.13(0.85-5.36) | 0.06 |
| *DRB1*1201* | 2 | 1.9 | 30 | 3.6 | 0.52(0.12-2.20) | 0.18 |
| *DRB1*1202* | 1 | 0.9 | 18 | 2.1 | 0.43(0.06-3.29) | 0.25 |
| *DRB1*1301* | 2 | 1.9 | 6 | 0.7 | 2.67(0.53-13.42) | 0.17 |
| ***DRB1*1302*** | 3 | 2.8 | 65 | 7.8 | **0.35(0.11-1.13)** | **0.03** |
| *DRB1*1403* | 0 | 0.0 | 11 | 1.3 | 0.34(0.02-5.79) | 0.27 |
| *DRB1*1405* | 2 | 1.9 | 17 | 2.0 | 0.93(0.21-4.09) | 0.29 |
| *DRB1*1406* | 3 | 2.8 | 13 | 1.6 | 1.85(0.52-6.61) | 0.17 |
| *DRB1*1454* | 6 | 5.7 | 26 | 3.1 | 1.88(0.75-4.67) | 0.08 |
| ***DRB1*1501*** | 21 | 19.8 | 67 | 8.0 | **2.85(1.66-4.89)** | **7.72E-05** |
| *DRB1*1502* | 11 | 10.4 | 70 | 8.4 | 1.27(0.65-2.49) | 0.48 |
| ***DRB1*1602*** | 3 | 2.8 | 2 | 0.2 | **12.2(2.02-73.9)** | **0.01** |

^a^alleles: risk alleles with frequencies of less than 1% in both cases and controls are omitted.

^b^OR: Odds ratio; 95% CI: lower and upper limits of confidence interval at 95%.

^c^P-values for allele frequency comparisons between cases and controls using the chi-square test.

| Supplementary Table S7. Association analysis of *HLA-DQB1* alleles with IMN in the first set | | | | | |  |
| --- | --- | --- | --- | --- | --- | --- |
| *HLA-DQB1* | Allele frequency | | | | | |
| alleles^a^ | IMN (2n=106) | | Control (2n=838) | | OR (95% CI)^b^ | P-value^c^ |
|  | No | % | No | % |  |  |
| *DQB1*0301* | 17 | 16.0 | 99 | 11.8 | 1.43(0.82-2.50) | 0.21 |
| *DQB1*0302* | 12 | 11.3 | 77 | 9.2 | 1.26(0.66-2.41) | 0.47 |
| *DQB1*0303* | 13 | 12.3 | 134 | 16.0 | 0.74(0.40-1.35) | 0.32 |
| ***DQB1*0401*** | **7** | **6.6** | **122** | **14.6** | **0.42(0.19-0.92)** | **0.01** |
| *DQB1*0402* | 1 | 0.9 | 26 | 3.1 | 0.30(0.04-2.22) | 0.14 |
| ***DQB1*0501*** | **3** | **2.8** | **63** | **7.5** | **0.36(0.11-1.16)** | **0.03** |
| *DQB1*0502* | 5 | 4.7 | 17 | 2.0 | 2.40(0.87-6.64) | 0.06 |
| *DQB1*0503* | 7 | 6.6 | 30 | 3.6 | 1.91(0.82-4.46) | 0.06 |
| *DQB1*0601* | 17 | 16.0 | 133 | 15.9 | 1.02(0.59-1.76) | 0.96 |
| ***DQB1*0602*** | **19** | **17.9** | **65** | **7.8** | **2.60(1.49-4.55)** | **5.12E-04** |
| *DQB1*0603* | 2 | 1.9 | 6 | 0.7 | 2.67(0.53-13.42) | 0.17 |
| ***DQB1*0604*** | **3** | **2.8** | **63** | **7.5** | **0.36(0.11-1.16)** | **0.03** |

^a^alleles: risk alleles with frequencies of less than 1% in both cases and controls are omitted.

^b^OR: Odds ratio; 95% CI: lower and upper limits of confidence interval at 95%.

^c^P-values for allele frequency comparisons between cases and controls using the chi-square test.

| *HLA-DPB1* | Allele frequency | |  |  |  |  |
| --- | --- | --- | --- | --- | --- | --- |
| alleles^a^ | IMN (2n=106) | | Control (2n=838) | | OR (95% CI)^b^ | P-value^c^ |
|  | No | % | No | % |  |  |
| *DPB1*0201* | 26 | 24.5 | 211 | 25.2 | 0.97(0.61-1.55) | 0.89 |
| *DPB1*0202* | 2 | 1.9 | 35 | 4.2 | 0.44(0.10-1.87) | 0.13 |
| *DPB1*0301* | 6 | 5.7 | 36 | 4.3 | 1.34(0.55-3.26) | 0.15 |
| ***DPB1*0401*** | 2 | 1.9 | 51 | 6.1 | **0.30(0.07-1.24)** | **0.04** |
| *DPB1*0402* | 7 | 6.6 | 83 | 9.9 | 0.64(0.29-1.43) | 0.08 |
| *DPB1*0501* | 45 | 42.5 | 322 | 38.4 | 1.19(0.79-1.79) | 0.41 |
| *DPB1*0601* | 2 | 1.9 | 5 | 0.6 | 3.21(0.62-16.76) | 0.15 |
| *DPB1*0901* | 12 | 11.3 | 66 | 7.9 | 1.50(0.78-2.87) | 0.22 |
| *DPB1*1301* | 0 | 0.0 | 12 | 1.4 | 0.31(0.02-5.29) | 0.24 |
| *DPB1*1401* | 2 | 1.9 | 10 | 1.2 | 1.60(0.34-7.38) | 0.25 |
| *DPB1*1901* | 2 | 1.9 | 5 | 0.6 | 3.21(0.62-16.76) | 0.15 |

^a^alleles: risk alleles with frequencies of less than 1% in both cases and controls are omitted.

Supplementary Table S8. Association analysis of *HLA-DPB1* alleles with IMN in the first set

^b^OR: Odds ratio; 95% CI: lower and upper limits of confidence interval at 95%.

^c^P-values for allele frequency comparisons between cases and controls using the chi-square test.

| Supplementary Table S9. Association between *HLA DRB1*15:01* and clinical outcome | | | |
| --- | --- | --- | --- |
| 50% increase of serum creatinine within one year | | |  |
|  | >50% increase | <50% increase | P-value |
| *DRB1*1501*(+) | 4 | 14 | N.S. |
| *DRB1*1501*(-) | 4 | 31 |  |
| Development of ESRD during follow-up period | | |  |
|  | ESRD | No ESRD | P-value |
| *DRB1*1501*(+) | 1 | 13 | N.S. |
| *DRB1*1501*(-) | 3 | 36 |  |
| Mortality during follow-up period | |  |  |
|  | non-survivor | survivor | P-value |
| *DRB1*1501*(+) | 3 | 13 | N.S. |
| *DRB1*1501*(-) | 7 | 30 |  |
| Improvement of proteinuria less than 1g/gCre | | |  |
|  | improve | not improve | P-value |
| *DRB1*1501*(+) | 9 | 8 | N.S. |
| *DRB1*1501*(-) | 12 | 24 |  |

ESRD: development to end stage renal disease.

| Supplementary Table S10. Association between *HLA DQB1*06:02* and clinical outcome | | | |
| --- | --- | --- | --- |
| 50% increase of serum creatinine within one year | | |  |
|  | >50% increase | <50% increase | P-value |
| *DQB1*0602*(+) | 4 | 12 | N.S. |
| *DQB1*0602*(-) | 5 | 32 |  |
| Development of ESRD during follow-up period | | |  |
|  | ESRD | No ESRD | P-value |
| *DQB1*0602*(+) | 1 | 15 | N.S. |
| *DQB1*0602*(-) | 4 | 33 |  |
| Mortality during follow-up period | |  |  |
|  | non-survivor | survivor | P-value |
| *DQB1*0602*(+) | 3 | 13 | N.S. |
| *DQB1*0602*(-) | 6 | 30 |  |
| Improvement of proteinuria less than 1g/gCre | | |  |
|  | improve | not improve | P-value |
| *DQB1*0602*(+) | 8 | 7 | N.S. |
| *DQB1*0602*(-) | 12 | 26 |  |

ESRD: development to end stage renal disease.
